# Effect of night light regimen on growth performance, antioxidant status and health of broiler chickens from 1 to 21 days of age

**DOI:** 10.5713/ajas.18.0525

**Published:** 2018-10-26

**Authors:** R. X. Zhao, C. H. Cai, P. Wang, L. Zheng, J. S. Wang, K. X. Li, W. Liu, X. Y. Guo, X. A. Zhan, K. Y. Wang

**Affiliations:** 1 Feed Science Institute, College of Animal Science, Zhejiang University, Hangzhou 310058, China; 2 Department of Biosystems Engineering, College of Biosystems Engineering and Food Science, Zhejiang University, Hangzhou 310058, China

**Keywords:** Night Light Regimen, Broiler Chickens, Growth Performance, Antioxidant Status, Health

## Abstract

**Objective:**

The study was conducted to evaluate the effects of night light regimen on growth performance, antioxidant status and health of Lingnan Yellow broiler chickens from 1 to 21 days of age.

**Methods:**

A completely randomized factorial design involved 2 photoperiods (constant lighting [CL], 24 L:0 D and intermittent lighting [INL], 17 L:3 D:1 L:3 D)×2 light intensities (10 lx and 30 lx). A total of one thousand six hundred and eighty 1-d-old Lingnan Yellow broiler chicks were randomly divided into 4 treatments with 6 replicates (70 birds per replicate). The experiment lasted for 21 d.

**Results:**

Photoperiods and light intensities had no effect on average daily gain, feed conversion ratio, and mortality of the broiler chickens (p>0.05). The INL had a significant effect on average daily feed intake (p<0.05) of broiler chickens compared with CL. Photoperiod and light intensity had an interactive effect on melatonin (MT) concentration (p<0.05). At CL, reducing light intensity increased MT concentration; INL birds had higher MT but MT concentration was not affected by light intensity. There was an interactive effect on glutathione peroxidase (GPx) and catalase (CAT) in serum and total antioxidant capability (T-AOC) in liver between photoperiod and light intensity. With the decrease of light intensity, the activities of GPx and CAT in serum and T-AOC in liver increased in CL group (p<0.05). Broiler chickens reared under INL had better antioxidant status and 10 lx treatments had higher activities of CAT in serum than 30 lx (p<0.05). Different photoperiods and light intensities had no effect on malondialdehyde. There was an interaction between photoperiod and light intensity on serum creatine kinase (CK) concentration (p<0.05). At CL, the elevated light intensity resulted in an increase in CK content; INL birds had lower CK concentration especially in low light intensity group. Besides, INL and low light intensity significantly reduced the concentration of serum corticosterone and heat shock protein 70 (p<0.05). Serum immunoglobulin M contents were increased in broiler chickens reared under the INL compared with CL group (p<0.05).

**Conclusion:**

Results above suggest that the night light regimen of INL and 10 lx could be beneficial to the broiler chickens from 1 to 21 days of age due to the better health status and electricity savings.

## INTRODUCTION

With the intensification and standardization of modern poultry industry, themicreenvironment has a great impact on the health and production performance of poultry. Lighting is one of the most important environmental factors affecting broiler performance and physical activity. It not only allows birds to establish rhythmicity and synchronize physiology, but also stimulates secretion patterns of several hormones controlling growth, maturation, and reproduction [1]. Therefore, broiler chickens reared under appropriate lighting regimens may have better performance as well as welfare advantages [2].

The different aspects of light, consisting of light intensity, duration and wavelength, have been studied in recent decades and more attention has been paid to light duration (photoperiod). Researches on different photoperiods mainly includes; continuous lighting (CL) and intermittent lighting (INL). The CL contains 24 h of light (L) and INL contains 2 or more light and dark (D) cycles in 24 h. Sönmez concluded that INL (12 L:3[1 L:3 D]) can improve the growth performance and reduce fast growth-related diseases, such as leg problems, sudden death syndrome and ascites of broiler chickens compared to the CL [3]. Broiler chickens reared under the INL (1 L:3 D cycles, repeated six times) compared to the CL had lower mortality and plasma T_3_ levels [4]. However, others like Renden et al [5] reported no difference in the growth performance of birds raised under INL when compared to those raised under CL.

Besides, light intensity is also one aspect of light management that could have important consequences for broiler behavior, performance and welfare. Reducing light intensity stimulated feed consumption and a subsequent body weight (BW) improvement, as compared with high-intensity lighting [6]. The results of Deep showed 1 lx light intensity treatment had a negative effect on broiler welfare as demonstrated by increased ulcerative footpad lesions and eye size compared with higher light intensity (10, 20, and 40 lx) [7]. Others found that there was no effect of light intensity on broiler chickens’ growth performance [8,9].

The results from our previous studies have demonstrated that compared to 16 L:2 D:1 L:2 D:1 L:2 D group, the 17 L:3 D:1 L:3 D group can enhance the antioxidant status of broiler chickens [10]. More studies are necessary to examine the effects of photoperiod, light intensity and their interaction on broiler chickens under commercial farming practice to assess the light program that maximizes growth performances with minimal negative impacts on broiler health. Thus, the study was conducted to evaluate the effects of night light regimen on growth performance, antioxidant status and health of broiler chickens from 1 to 21 days of age to determine the proper night light regimen for commercial raising.

## MATERIALS AND METHODS

The project was conducted under the supervision of Zhejiang University Animal Care and Use Committee (Hangzhou, China), which has adopted animal care and use guidelines governing all animal use in experimental producers [11–13].

### Experimental design and treatments

To examine the effects of night light regimen on broiler chickens, a completely randomized design involving 2 photoperiods×2 light intensities was used in this experiment. The 2 photoperiods were CL (24 L:0 D) and INL (17 L:3 D:1 L:3 D). During the daytime we used natural light and the illumination was provided by incandescent bulbs at night. Automatic timers (KG316T, Xiangyang Electronic Co., Ltd., Zhejiang, China) were used for different lighting photoperiods. The intermittent cycle began at 20:00 and finished at 03:00 for INL. The 2 light intensities were 10 lx and 30 lx. Light intensity was measured along a horizontal plane at 25 cm above the litter using a light meter (VICTOR 1010C, Shenzhen City Station Win Technology Co., Ltd., Guangdong, China) 3 times each week. Therefore, there were a total of 4 treatments (CL/10 lx, CL/30 lx, INL/10 lx, INL/30 lx) in this experiment.

### Animals and diets

A total of one thousand six hundred and eighty 1-d-old Lingnan Yellow broiler chicks were obtained from a local commercial hatchery (Zhejiang Yixing Industry Co. Ltd., Jiaxing, China) and randomly divided into four treatments with 6 replicates of 70 birds per replicate based on BW. Four groups of broiler chickens were housed in four rooms with similar environments except the light regimen. The room temperature was set initially at 33°C and gradually reduced by 3°C per week. The birds were provided with clean water and fed ad-libitum during the experimental period. Birds were reared in floor pens cover with fresh wood shavings. The experiment lasted for 21 d.

A corn-soybean meal basal diet was formulated to meet the nutrient requirement guidelines of the NRC [14]. The ingredients and nutrient content of the maternal basal diets are shown in Table 1 [14].

### Sample collection and preparation

At the end of the 21 d experiment, 4 birds (2 males and 2 females) were randomly selected per replicate from each treatment. Feed was withdrawn from the birds approximately 12 h before slaughter. Blood samples (5.0 mL per birds) were collected from the main wing vein and placed in coagulant tubes. Then, the birds were slaughtered to obtain the liver (part of the liver left lobe). Serum was separated by centrifugation at 4°C, 3,000 rpm/min for 10 min. All samples were frozen directly in liquid nitrogen and stored at −80°C for further analysis.

### Growth performance

The BW was measured at the beginning of the experiment (1 d) and on 21 d. Both BW and feed intake (FI) were measured for the calculation of average daily feed intake (ADFI), average daily gain (ADG) and feed conversion ratio (FCR). Mortality was recorded daily as it occurred. The FCR calculated as the total amount of feed consumed divided by the total BW was adjusted for the number and weight of the dead birds during the experimental period.

### Serum melatonin concentration

Serum melatonin (MT) was measured by enzyme-linked immunosorbent assay (ELISA) and its concentration expressed as pg/mL. The MT test kit utilizes the principles of competition.

### Antioxidant status

Antioxidant indexes were assayed according to the prescribed protocol using commercial kits. Serum and liver were analyzed for the activities of glutathione peroxidase (GPx), total antioxidant capability (T-AOC), catalase (CAT), and malondialdehyde (MDA).

The GPx activity was assessed using a GPx kit, which was developed based on the analysis of reduced glutathione (GSH) in the enzymatic reaction. One unit of enzyme activity represents a decrease in GSH concentration of 1 μmol/mg protein per minute after subtraction of non-enzymatic mode at 37°C [15].

2,2′-Amino-di(2-ethyl-benzothiazoline sulphonic acid-6) ammonium salt (ABTS) is oxidized to a green ABTS^+^ under the action of a suitable oxidizing agent. The antioxidant inhibits the production of ABTS^+^. The absorbance of ABTS^+^ is measured at 405 nm or 734 nm to determine and calculate the T-AOC of the sample.

The reaction of CAT to decompose H _2_O_2_ can be quickly stopped by the addition of ammonium molybdate, and the remaining H_2_O_2_ reacts with ammonium molybdate to produce a pale-yellow complex. By measuring the amount of change, the activity of CAT can be calculated.

The MDA level was determined via the method of Yagi [16]. Its principle is based on the intensity of the color after treatment of the sample with thiobarbituric acid [16].

The total protein concentration of liver was measured by Lowry method using commercial kit [17]. All kits were manufactured by Nanjing Jiancheng Bioengineering Institute (Nanjing, China).

### Anti-stress capacity

Serum was analyzed for creatine kinase (CK), corticosterone (CORT) and heat shock protein 70 (HSP70) by using commercial kits manufactured by Nanjing Jiancheng Bioengineering Institute (China).

The CK catalyzes the activation of adenosine triphosphate and creatine to produce creatine phosphate. The addition of ammonium molybdate produces phosphomolybdic acid, which can be further reduced to molybdenum blue, and the activity of the enzyme can be calculated from the amount of inorganic phosphorus produced.

The CORT and HSP _70_ was measured by ELISA. Manufacturer’s recommendations were followed during the assay.

### Immunity

Concentrations of immunoglobulin A (IgA), IgM, and IgG in serum were determined by turbidimetry with commercial kits manufactured by Nanjing Jiancheng Bioengineering Institute (China). This test gave the total level of non-specific IgA, IgM, and IgG.

The IgA, IgM, and IgG in serum and the specific IgA, IgM, and IgG antibody in the reagent form an antigen-antibody complex resulting in turbidity, and its turbidity is proportional to the amount of IgA, IgM, and IgG in the serum in the presence of a certain amount of antibody. By measuring the absorbance value at a specific wavelength, the IgA, IgM, and IgG content in the serum can be calculated by referring to the calibration curve.

### Statistical analysis

Replicate was considered as the experimental unit. Data were analyzed as a 2×2 (photoperiods×light intensities) factorial arrangement of treatments by two-way analysis of variance with a model including the main effects of photoperiods, light intensities and their interaction using the general linear model procedure of the SPSS 19.0. The values were expressed as means ±standard error and statistical significance was set at p<0.05.

## RESULTS

### Growth performance

The results of the effects of night light mode on ADG, ADFI, FCR and mortality are presented in Table 2. Photoperiods and light intensities had no effect on ADG, FCR, and mortality of the broiler chickens (p>0.05). The INL had significant effect on ADFI (p<0.05) of broiler chickens compared with CL.

### Serum melatonin concentration

As shown in Table 3. Photoperiod and light intensity had an interactive effect on MT concentration (p<0.05). At CL, reducing light intensity increased MT concentration; INL birds had higher MT but MT concentration was not affected by light intensity.

### Antioxidant status

Table 4 and Table 5 show an interactive effect on GPx and CAT in serum and T-AOC in liver between photoperiod and light intensity. With the decrease of light intensity, the activities of GPx and CAT in serum and T-AOC in liver increased in CL group (p<0.05). Broiler chickens reared under INL had better antioxidant status and 10 lx treatments had higher activities of CAT in serum than 30 lx (p<0.05). Different photoperiods and light intensities had no effect on MDA.

### Anti-stress capacity

Both photoperiod and light intensity affected (p<0.05) anti-stress capacity (Table 6). There was an interaction between photoperiod and light intensity on serum CK concentration (p<0.05). At CL, the elevated light intensity resulted in an increase in CK content; INL birds had lower CK concentration especially in low light intensity group. Besides, INL and low light intensity significantly reduced the concentration of serum CORT and HSP_70_ (p<0.05).

### Immune functions

In Table 7, photoperiod and light intensity and their interaction did not affect (p>0.05) concentrations of IgG and IgA, but serum IgM contents were increased in broiler chickens reared under the INL compared with CL group (p<0.05).

## DISCUSSION

Our results revealed that different photoperiods and light intensities had no effect on ADG, FCR, and mortality. Similar results have been reported by Bayram, who found that there were no significant differences between 16 L:8 D group and 24 L:0 D group in BW and FCR at 6 wk [18]. Ahmad et al [19] reported that there was no effect of various light intensities on overall growth performance of broiler chickens. Olanrewaju et al [20] compared 5 lx to 20 lx and did not report any significant difference in growth performance. However, Olanrewaju stated that broiler chickens subjected to a short/non-intermittent photoperiod showed the significantly lowest BW, body weight gain, and FI compared with broiler chickens reared under long/continuous (23 L:1 D) and regular/intermittent (2 L:2 D) photoperiods [21]. We found INL group had higher ADFI than CL. An increased feed consumption was also noted in broiler chickens provided 2.7 lx instead of 21.5 lx [22]. Our results showed that there was no interaction of photoperiod and light intensity on broiler chickens growth performance, this finding agreed with the results of Olanrewaju, who reported no interaction effect between 3 light intensities (10, 5.0, and 0.5 lx) and 3 photoperiods (23:1 D, 2 L:2 D, and 8 L:16 D) on broiler chickens growth performance [20].

The MT is a neuro-hormone secreted by the pineal gland in vertebrates during darkness. Data obtained from our experiment exhibited that INL can lead to higher MT concentration compared to CL, but light intensity had no significant effect on it. Previous studies also reported that, broiler chickens reared under CL were more severely deficient in MT than those reared under INL [22]. Özkan et al [23] found that broiler chickens reared at 16 L:8 D had significantly higher plasma MT concentration than CL. This can be explained by Illnerová et al [24], who revealed that the duration of MT release is proportional to the length of the dark phase. In addition, Zheng et al [25] agree with our results that there was no significant difference among 4 treatments (5, 10, 20, 30 lx) in serum MT concentration of broiler chickens. Illumination could decrease MT concentration in systematic circulation when light intensity is above 500 lx in Syrian hamsters, sheep and monkeys [26–28]. Because the secretion of MT is only inhibited when the light intensity reaches a certain intensity, light intensity used in the present study may have not been sufficient enough to cause an obvious difference.

In the process of raising broiler chickens, adjusting the light in the environment can influence the secretion of MT and indirectly regulate the antioxidant status of the body. Apart from its direct free radical scavenging properties, MT plays a crucial role in the antioxidant system because it can promote the production of GPx, the main antioxidant enzyme in the brain [29,30]. The enzymes of GPx and CAT play an important role in scavenging oxygen free radicals and protecting cells from damage due to oxidation by free radicals and lipid peroxides. Tyssandier et al [31] reported that T-AOC can be used as a comprehensive index to measure the antioxidant status of the body. The MDA is one of the final products of polyunsaturated fatty acids peroxidation in the cells. Its level is commonly known as a marker of oxidative stress and the antioxidant status [32]. A study by Guo et al [33] showed 12 L:12 D decreased serum MDA and enhanced oxidant-antioxidant balance compared with 23 L:1 D, 20 L:4 D, and 16 L:8 D. Zheng et al [10] demonstrated that 3 D:1 L significantly enhanced the total T-AOC and GSH-Px. These findings agree with our results that INL groups had better antioxidant status compared to CL groups. Zheng et al [25] reported that low light intensity of 5 lx significantly enhanced T-AOC and activity of GPX of 21d broiler chickens both in serum and liver. Similar results were observed in our study. Besides, there was an interactive effect on GPx and CAT in serum and T-AOC in liver between photoperiod and light intensity. Researches about the interactive effect of photoperiod and light intensity on antioxidant status are limited.

Previous studies have shown that the anti-stress properties of MT make it possible to counteract the HPA axis dysfunction induced by exogenous glucocorticoids, and then reduce the stress caused by the rise of adreno-cortico-tropic-hormone, CORT, and other hormones [34]. Creatine kinase, which is an intracellular enzyme, will be released into the bloodstream, resulting in increased serum CK concentrations. The content of CK is related to the production of free radicals in the body, but MT can reduce the content of CK in the blood by scavenging free radicals. The HSP_70_ is one of the most conservative and important members of heat stress proteins. The level of HSP_70_ is low in normal cells and can be significantly elevated under stress conditions. Our results revealed that INL and low light intensity can minimize the stress compared with CL and high light intensity. This is supported by the finding of Olanrewaju and Buckland who reported that birds in CL group had a higher CORT concentration than INL group in their serum [1,35]. However, researches about the effects of light intensity on anti-stress capability are limited.

When animals are stimulated by an antigen, an immuno globulin that specifically binds to the antigen in the body is produced. As common immunoglobulin, IgA, IgM, and IgG are of vital importance in humoral immunity. Akbulut found exogenous MT can increase IgG1 and IgM responses of aged rats [36]. This showed that MT can raise the levels of immunoglobulin in serum to some extent, which can be the reason that serum IgM contents were increased (p<0.05) in broiler chickens reared under the INL compared to CL group in our experiment. Guo et al [33] reported that plasma IgG in the 12 L:12 D group was higher than in the CL group. Blatchford observed that there was no difference between different light intensities for most immune parameters, which was in accordance with our results [8].

## CONCLUSION

Broiler chickens reared under INL and 10 lx of light intensity can increase MT concentration and improve antioxidant status, anti-stress capacity and immune function. Results above suggest that the night light regimen of INL and 10 lx could be beneficial to broiler chickens from 1 to 21 days of age due to the better health status and electricity saving issue.

## Figures and Tables

**Table 1:** Ingredients and nutrient content of the basal diets

Item	1 to 21 d
Ingredient (%)	
Corn	54.70
Wheat	5.00
Soybean meal	29.00
Corn gluten meal	6.00
Soy oil	1.00
Salt	0.30
CaHPO_4_	1.70
Limestone	1.30
Vitamin-mineral premix1)	1.00
Composition (%)	
ME2) (MJ/kg)	12.17
Crude protein	20.96
Lys	1.10
Met	0.50
Met+Cys	0.85
Calcium	0.99
Total phosphorus	0.66

1) The vitamin-mineral premix supplied the followings per kilogram of diet: vitamin A, 9,600 IU; vitamin D_3_, 2,700 IU; vitamin E, 36 mg; vitamin K_3_, 3.0 mg; vitamin B_1_, 3.0 mg; vitamin B_2_, 10.5 mg; vitamin B_6_, 4.2 mg; vitamin B_12_, 0.03 mg; folic acid, 1.5 mg; nicotinamide, 60 mg; D-calcium pantothenate, 18 mg; biotin, 0.225 mg; choline chloride, 1,000 mg; Fe, 80 mg; Cu, 8.0 mg; Mn, 80 mg; Zn, 60 mg; I, 0.35 mg; Se, 0.15 mg.

2) The metabolizable energy (ME) was calculated from data provided by Feed Database in China.

**Table 2:** Effect of night light mode on growth performance of broilers

Photoperiod	Light intensity	ADG (g)	ADFI (g)	FCR (g/g)	Mortality (%)
CL1)	30 lx	22.04	34.41	1.56	0.71
	10 lx	22.10	34.31	1.55	1.19
INL2)	30 lx	22.14	35.35	1.60	1.43
	10 lx	22.83	36.76	1.61	0.95
SEM		0.196	0.363	0.012	0.262
CL1)	-	22.07	34.36	1.56	0.95
INL2)	-	22.49	36.05	1.60	1.19
-	30 lx	22.09	34.88	1.58	1.07
-	10 lx	22.47	35.54	1.58	1.07
p-value	Photoperiod	0.320	0.009	0.524	0.659
	Light intensity	0.370	0.219	0.222	1.000
	Photoperiod×light intensity	0.453	0.164	0.831	0.815

Values are means of six replicates (n = 6).

ADG, average daily gain; ADFI, average daily feed intake; FCR, feed conversion ratio; CL, constant lighting; INL, intermittent lighting.

1) CL = 24 L:0 D.

2) INL = 17 L:3 D:1 L:3 D.

**Table 3:** Effects of night light mode on serum melatonin concentration of broilers (pg/mL)

Photoperiod	Light intensity	Melatonin
CL1)	30 lx	55.25^c^
	10 lx	57.35^b^
INL2)	30 lx	58.18^ab^
	10 lx	59.21^a^
SEM		0.287
CL1)	-	56.30
INL2)	-	58.70
-	30 lx	56.71
-	10 lx	58.28
p-value	Photoperiod	<0.001
	Light intensity	0.134
	Photoperiod×light intensity	<0.001

Values are means of six replicates (n = 6).

CL, constant lighting; INL, intermittent lighting; SEM, standard error of the mean.

1) CL = 24 L:0 D.

2) INL = 17 L:3 D:1 L:3 D.

a,b,c Means in a column with different superscripts are significantly different (p<0.05).

**Table 4:** Effects of night light mode on serum antioxidant status of broilers

Photoperiod	Light intensity	GPx (U/mL)	T-AOC (U/mL)	CAT (U/mL)	MDA (nmol/mL)
CL1)	30 lx	3,939^b^	8.11	2.38^c^	3.70
	10 lx	5,804^a^	8.24	3.17^ab^	3.99
INL2)	30 lx	5,964^a^	9.97	2.89^b^	3.73
	10 lx	6,087^a^	8.50	3.37^a^	3.43
SEM		145,870	0.306	0.092	0.082
CL1)	-	4,871	8.17	2.77	3.85
INL2)	-	6,025	9.23	3.13	3.58
-	30 lx	4,951	9.04	2.64	3.71
-	10 lx	5,945	8.37	3.27	3.71
p-value	Photoperiod	<0.001	0.080	0.023	0.100
	Light intensity	<0.001	0.261	0.016	0.989
	Photoperiod×light intensity	<0.001	0.182	<0.001	0.073

Values are means of six replicates (n = 6).

GPx, glutathione peroxidase; T-AOC, total antioxidant capability; CAT, catalase; MDA, malondialdehyde; CL, constant lighting; INL, intermittent lighting; SEM, standard error of the mean.

1) CL = 24 L:0 D.

2) INL = 17 L:3 D:1 L:3 D.

a,b,c Means in a column with different superscripts are significantly different (p<0.05).

**Table 5:** Effects of night light mode on liver antioxidant status of broilers

Photoperiod	Light intensity	GPx (U/mgprot)	T-AOC (U/mgprot)	CAT (U/mgprot)	MDA (nmol/mgprot)
CL1)	30 lx	31.62	0.69^b^	2.97	1.04
	10 lx	30.89	1.31^a^	3.14	1.09
INL2)	30 lx	33.07	1.34^a^	3.79	1.15
	10 lx	30.06	1.45^a^	3.56	1.12
SEM		1.124	0.058	0.141	0.039
CL1)	-	31.25	1.00	3.05	1.06
INL2)	-	31.57	1.39	3.68	1.13
-	30 lx	32.34	1.02	3.38	1.09
-	10 lx	30.48	1.38	3.35	1.10
p-value	Photoperiod	0.893	<0.001	0.028	0.394
	Light intensity	0.421	<0.001	0.921	0.885
	Photoperiod×light intensity	0.622	0.003	0.465	0.572

Values are means of six replicates (n = 6).

GPx, glutathione peroxidase; T-AOC, total antioxidant capability; CAT, catalase; MDA, malondialdehyde; CL, constant lighting; INL, intermittent lighting; SEM, standard error of the mean.

1) CL = 24 L:0 D.

2) INL = 17 L:3 D:1 L:3 D.

a,b Means in a column with different superscripts are significantly different (p<0.05).

**Table 6:** Effects of night light mode on serum anti-stress capacity of broilers

Photoperiod	Light intensity	CK (U/L)	CORT (ng/mL)	HSP_70_ (pg/mL)
CL1)	30 lx	35.99^a^	24.70	48.32
	10 lx	22.63^b^	18.21	31.44
INL2)	30 lx	21.57^b^	14.79	35.59
	10 lx	15.63^c^	12.46	18.10
SEM		1.436	1.156	1.895
CL1)	-	29.31	21.46	39.83
INL2)	-	18.60	13.62	28.81
-	30lx	28.78	19.75	43.87
-	10lx	19.13	15.33	24.77
p-value	Photoperiod	<0.001	<0.001	0.002
	Light intensity	<0.001	0.022	<0.001
	Photoperiod×light intensity	0.033	0.269	0.486

Values are means of six replicates (n = 6).

CK, creatine kinase; CORT, corticosterone; HSP_70_, heat shock protein 70; CL, constant lighting; INL, intermittent lighting; SEM, standard error of the mean.

1) CL = 24 L:0 D.

2) INL = 17 L:3 D:1 L:3 D.

a,b,c Means in a column with different superscripts are significantly different (p<0.05).

**Table 7:** Effects of night light mode on serum contents of immunoglobulins of broilers

Photoperiod	Light intensity	IgG (ng/mL)	IgM (ng/mL)	IgA (ng/mL)
CL1)	30 lx	311.4	180.4	6.25
	10 lx	314.0	179.1	6.26
INL2)	30 lx	314.1	184.5	6.27
	10 lx	313.6	200.6	6.33
SEM		0.376	3.264	0.012
CL1)	-	312.7	179.7	6.26
INL2)	-	313.8	192.6	6.30
-	30lx	312.7	182.4	6.26
-	10lx	313.8	189.9	6.30
p-value	Photoperiod	0.107	0.044	0.069
	Light intensity	0.139	0.236	0.103
	Photoperiod×light intensity	0.133	0.165	0.245

Values are means of six replicates (n = 6).

Ig, immunoglobulin; CL, constant lighting; INL, intermittent lighting; SEM, standard error of the mean.

1) CL = 24 L:0 D.

2) INL = 17 L:3 D:1 L:3 D.
